# Induction of Thymus Atrophy and Disruption of Thymocyte Development by Fipronil through Dysregulation of IL-7-Associated Genes

**DOI:** 10.1021/acs.chemrestox.4c00060

**Published:** 2024-08-14

**Authors:** Jui-Fang Kuo, Hsin-Ying Wu, Chun-Wei Tung, Wei-Hsiang Huang, Chen-Si Lin, Chia-Chi Wang

**Affiliations:** † School of Veterinary Medicine, National Taiwan University, Taipei 106, Taiwan; ‡ Laboratory Animal Center, National Health Research Institutes, Miaoli County 350, Taiwan; § Institute of Biotechnology and Pharmaceutical Research, National Health Research Institutes, Miaoli County 350, Taiwan; ∥ Graduate Institute of Molecular and Comparative Pathobiology, National Taiwan University, Taipei 106, Taiwan

## Abstract

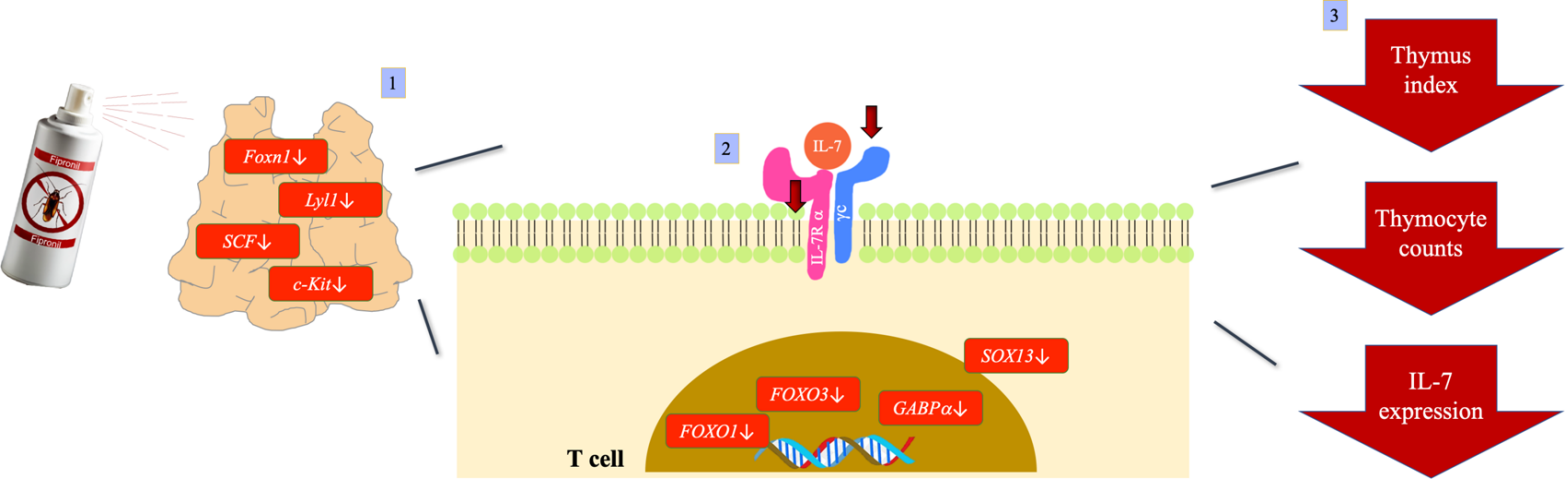

The susceptibility of the immune system to immunotoxic chemicals is evident, particularly in the thymus, a vital primary immune organ prone to atrophy due to exposure to toxicants. Fipronil (FPN), a widely used insecticide, is of concern due to its potential neurotoxicity, hepatotoxicity, and immunotoxicity. Our previous study showed that FPN disturbed the antigen-specific T-cell functionality *in vivo*. As T-cell lineage commitment and thymopoiesis are closely interconnected with the normal function of the T-cell-mediated immune responses, this study aims to further examine the toxic effects of FPN on thymocyte development. In this study, 4-week-old BALB/c mice received seven doses of FPN (1, 5, 10 mg/kg) by gavage. Thymus size, medulla/cortex ratio, total thymocyte counts, double-positive thymocyte population, and IL-7-positive cells decreased dose-dependently. IL-7 aids the differentiation of early T-cell precursors into mature T cells, and several essential genes contribute to the maturation of T cells in the thymus. *Foxn*1 ensures that the thymic microenvironment is suitable for the maturation of T-cell precursors. *Lyl*1 is involved in specifying lymphoid cells and maintaining T-cell development in the thymus. The *c-Kit/SCF* collaboration fosters a supportive thymic milieu to promote the formation of functional T cells. The expression of *IL-*7, *IL-*7*R*, *c-Kit*, *SCF*, *Foxn*1, and *Lyl*1 genes in the thymus was significantly diminished in FPN-treated groups with the concordance with the reduction of IL-7 signaling proteins (IL-7, IL-7R, c-KIT, SCF, LYL1, FOXO3A, and GABPA), suggesting that the dysregulation of T-cell lineage-related genes may contribute to the thymic atrophy induced by FPN. In addition, FPN disturbed the functionality of thymocytes with an increase of IL-4 and IFN-γ production and a decrease of IL-2 secretion after T-cell mitogen stimulation *ex vivo*. Collectively, FPN significantly deregulated genes related to T-cell progenitor differentiation, survival, and expansion, potentially leading to impaired thymopoiesis.

## Introduction

Fipronil (FPN) is a Class II moderately hazardous pesticide.^1−5^ Its widespread use raised concerns regarding its impact on nontarget organisms and various organ systems. Cumulative pollution and toxicity have been found in the natural ecosystem as well as in beneficial insects like bees and dragonflies.^6^ Accordingly, the European Union imposed a ban on its use in 2013.^7,8^ Furthermore, FPN pesticides are also strictly prohibited for use in food-producing animals in the EU and other countries. Although FPN has been considered a low-toxicity pesticide, several studies have shown that FPN has neurotoxic, hepatotoxic, and reproductive effects on nontarget organisms, including mammals, birds, and aquatic species.^9−12^

Humans are exposed to FPN in a variety of ways, including occupational exposure (155/159 workers),^13^ unintentional exposure (FPN and its metabolite can be detected within 25% of sampling serum, *n* = 96),^14−16^ self-poisoning (6 cases),^17^ or consumption of contaminated food or drinking water.^15,18−21^ Approximately 40% of FPN residues in American households were detected through contact with pets treated with FPN-containing products.^14,22^ With a surge in adverse outcome reports of pets treated with FPN, the U.S. Environmental Protection Agency (EPA) has intensified its examination of spot-on insecticides containing FPN. It has been reported that FPN could be rapidly absorbed through the gastrointestinal tract,^23,24^ and its more toxic metabolite, FPN sulfone, exhibits a persistent accumulation in the body for up to 7 days in acute self-poisoning humans.^25^ Fipronil sulfone can be detected in the serum of newborns, implying that if women are exposed to FPN during pregnancy, its metabolites can transfer through the placenta to newborn infants and lead to adverse effects on thyroid function and Apgar score.^26^ Therefore, the toxic effects of exposure to FPN pesticides remain a significant public health concern and require mechanistic studies of their potential hazards to vertebrates.

The immune system displays heightened sensitivity to toxic responses induced by various chemicals, particularly affecting the thymus, which is prone to atrophy upon exposure to compounds like immunosuppressive drugs and environmental chemicals.^27−31^ Serving as a vital immune organ, the thymus plays a pivotal role in coordinating the maturation, selection, and differentiation of the majority of naive T cells.^32^ Despite its functionality declining with age, the thymus remains crucial for T-cell-repertoire reconstitution, ensuring immune responses in diverse situations until late adulthood.^28^ Potential threats to immune function arise from the impact of compounds that induce atrophy on the thymus, making the thymus a sensitive indicator of the immunotoxicity of toxicants.

The process of T-cell lineage commitment necessitates collaboration between thymocytes and thymic epithelial cells (TECs) within the thymic microenvironment. This intricate interaction is tightly regulated by several transcription factors and the IL-7 signaling pathway.^33^ In T-cell development, thymic IL-7, produced by TECs, binds to IL-7R on immature T lymphoid progenitor cells to further promote their proliferation, differentiation, and survival.^34,35^ In addition, TECs may release stem cell factor (SCF) to drive thymocyte expansion at several stages through activation of Kit receptors. Disruption of Foxn1 may impede the maturation of TECs and indirectly result in the loss of intrathymic T-cell development and the manifestation of immunodeficiency.^36^*Lyl*1 is involved in the regulation of lymphoid specification.^37^ These genes play crucial roles in shaping the microenvironment required for T-cell development and facilitating progression through various stages of thymopoiesis.

Recent studies revealed the immunotoxic effects of FPN on mammals. The oral administration of 10% LD_50_ (9.7 mg/kg) FPN to rats resulted in histopathological alterations in the spleen and thymus tissues. Additionally, an increase in proinflammatory cytokines and antibodies in the serum suggested that FPN triggered allergic and inflammatory responses in male rats, concurrently impairing lymphocyte function.^38^ In human lymphocytic Jurkat cells, FPN demonstrated a direct reduction in the synthesis of IL-2 and IFN-γ, indicating a potential direct impact on T cells even at noncytotoxic concentrations.^39^ In our previous study, we demonstrated that FPN treatment disturbed antigen-specific immune responses through dysregulation of GABAergic genes *in vivo.*(40) Despite the disclosed adverse effects of FPN on the immune system, limited knowledge exists regarding how FPN modulates T-cell lineage commitment and maturation in the thymus. Regarding the adverse effects of FPN on mature T-cell function, this study aims to further examine the effects of FPN on thymocyte development *in vivo*.

## Materials and Methods

### Reagents

Fipronil (FPN, 97%) was obtained from Tokyo Chemical Industry Co., Ltd. (Tokyo, Japan). RPMI 1640 medium (catalog no. SH30027.02) was purchased from Hyclone (UT). Fetal bovine serum (FBS, cat. no. 10437-028) and cell culture reagents were purchased from GIBCO BRL (MD) and GE Healthcare (Chicago, IL). Reagents for enzyme-linked immunosorbent assay (ELISA) analysis were provided by BD Biosciences (San Jose, CA). All other reagents were acquired from Sigma (MO) unless otherwise specified.

### Experimental Animals

The BALB/c mice (3 weeks old; weighing 12–14 g) were supplied by the BioLASCO Experimental Animal Center (BioLASCO, Taipei, Taiwan). After their arrival, the mice were randomly assigned to groups and weighed for randomization. To minimize initial weight differences within each category, the mice were then categorized based on their combined weight into five groups. Individual housing was provided, maintaining controlled conditions, including a 12-h light/dark cycle, temperature (22 ± 2 °C), humidity (40 ± 15%), and unrestricted access to standard laboratory food and water *ad libitum*. Animal experiments were conducted following the guidelines of the Institutional Animal Care and Use Committee of the National Taiwan University (IACUC Approval No: NTU108-EL-00026).

### Protocol of Animal Experiment

**Figure 1:**
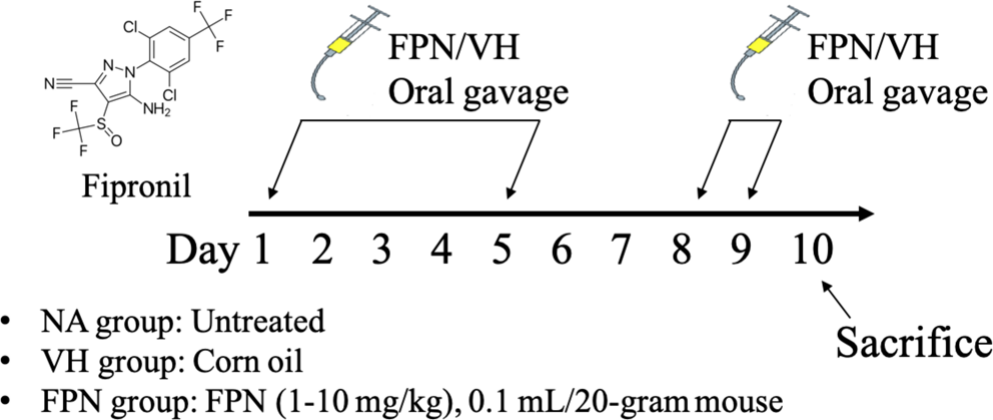
Protocol for fipronil (FPN) administration. Mice were randomly divided into the following groups: naïve (NA), vehicle-treated (VH), and FPN-treated group. The dosing regimen for FPN administration is described in Materials and Methods.

Following a 1-week acclimatization period, 4-week-old mice (5 animals/group) were randomly assigned to five groups, including no treatment groups (naïve; NA), vehicle control group (VH; corn oil), and oral gavage with fipronil (FPN) at doses of 1, 5, or 10 mg/kg suspended in corn oil for a total of seven doses (Figure 1). Based on previous studies, 10 mg/kg of FPN (equivalent to 1/10 of the oral LD_50_ in mice) was chosen to minimize the risk of acute toxicity and mortality while still inducing subchronic toxic effects over a seven-dose treatment period.^1,40^ The other doses of 1 mg/kg (1/100 LD_50_) and 5 mg/kg (1/20 LD_50_) were selected to demonstrate dose-related effects of FPN. On day 10, the mice were euthanized, and their thymus was harvested for studying the systemic immune responses. Since the mice needed to be monitored for clinical changes following exposure to FPN, the experimenter could not be blinded to whether the animals were exposed to FPN or corn oil.

### Thymocyte Isolation and Culture

The thymus was aseptically removed from mice, washed, and then processed into a single-cell suspension. The culture medium was RPMI 1640 medium supplemented with 5% heat-inactivated FBS, 100 U/mL penicillin, and 100 μg/mL streptomycin. In all cases, thymocytes were cultured at 37 °C in 5% CO_2_. Thymocyte counts were determined using a Moxi Z Mini Automated Cell Counter (ORFLO, ID).

### Thymus Index

The thymus from each mouse (*n* = 20 in each group) was aseptically dissected and weighed immediately upon euthanasia. The thymus index was calculated as the thymus weight (mg) divided by the body weight (g) of the mouse.

### Flow Cytometric Analysis for Cellularity of Thymocytes

The primary thymocytes were stained with rat antimouse CD4 conjugated with FITC (BD Biosciences, San Jose, CA), and/or rat antimouse CD8 conjugated with PE-Cy5 (BD Biosciences, San Jose, CA), and/or rat antimouse TCRαβ conjugated with APC (BioLegend, San Diego, CA), and/or rat antimouse TCRγδ conjugated with PE (BioLegend, San Diego, CA) antibodies in phosphate-buffered saline (PBS) containing 2% FBS. Appropriate rat antimouse antibodies were applied as the isotype control. For each sample, 10,000 events were collected and measured by a flow cytometer (BD FACSCalibur, San Jose, CA). Data was analyzed by Flowjo 10.4 software (FlowJo LLC, Ashland, OR).

### Cytotoxicity Assay

The cytotoxicity of FPN to thymocytes was determined by the 3-(4,5-dimethylthiazol-2-yl)-2,5-diphenyltetrazolium bromide (MTT) assay as previously described.^41^ Thymocytes (6 × 10^6^ cells/mL) were cultured in triplicate (100 μL/well) in 96-well culture plates followed by stimulation with concanavalin A (ConA) for 44 h. Following stimulation, a stock solution of MTT (5 mg/mL) was added and incubated for 4 h. Subsequently, the resultant formazan was dissolved with the dimethyl sulfoxide (DMSO). The absorbance was read by an ELISA microplate reader (SpectraMax M5Microplate Reader, Molecular Devices LLC, San Jose, California) at an OD_570nm_, with OD_630nm_ utilized as a background reference for accurate measurements.

### Measurement of Cytokines by Enzyme-Linked Immunosorbent Assay (ELISA)

Thymocytes (6 × 10^6^ cells/mL) were cultured in quadruplicate in a 48-well culture plate (0.3 mL/well). The culture supernatant with concanavalin A (ConA) stimulation (48 h) was collected to examine the level of IL-2, IL-4, and IFN-γ by ELISA kit (BD Biosciences, San Jose, CA) as previously described.^40^ The optical density was measured at OD_450nm_ by using an ELISA microplate reader (SpectraMax M5Microplate Reader, Molecular Devices LLC, San Jose, CA).

### RNA Isolation and Quantitative Polymerase Chain Reaction (qPCR)

Total RNA from thymus tissue and isolated thymocytes (stimulated by ConA for 24 h) were homogenized using TRIzol reagent and isolated using the GENEzol Pure Kit (Geneaid Biotech Ltd., New Taipei City, Taiwan), following the manufacturer’s instructions. Subsequently, cDNA was prepared, and quantitative PCR (qPCR) was conducted as previously described.^40^ Expression levels of target genes were determined by using the ΔΔ*C*_t_ method and normalized to the HRPT mRNA content. The primers for the target genes used in this study are listed in Table 1.

**Table 1:** List of Quantitative PCR Primers

gene name	primers (5′ to 3′)
IL-7	F: TCTGCTGCCTGTCACATCATC
	R: GGACATTGAATTCTTCACTGATATTCA
IL-7 receptor	F: CACAGCCAGTTGGAAGTGGATG
	R: GGCATTTCACTCGTAAAAGAGCC
SCF	F: CCCTGAAGACTCGGGCCTA
	R: CAATTACAAGCGAAATGAGAGCC
c-Kit	F: GAGTTCCATAGACTCCAGCGTC
	R: AATGAGCAGCGGCGTGAACAGA
GABPα	F: CCGCTACACCGACTACGATT
	R: ACCTTCATCACCAACCCAAG
FOXO1	F: CTACGAGTGGATGGTGAAGAGC
	R: CCAGTTCCTTCATTCTGCACTCG
FOXO3	F: CCTACTTCAAGGATAAGGGCGAC
	R: GCCTTCATTCTGAACGCGCATG
*Foxn*1	F: TGACGGAGCACTTCCCTTAC
	R: GACAGGTTATGGCGAACAGAA
*Lyl*1	F: CAGCTAACTGCCTTGGGAAG
	R: CCAGCTCACTATGGCTTGGT
*SOX*13	F: GATGCCACCAACGCTAAAGC
	R: TTGCGGTTGAAGTCCAGGC
*HPRT*	F: TCAGTCAACGGGGGACATAAA
	R: GGGGCTGTACTGCTTAACCAG

### Histological Examination

Formalin-fixed tissue sections of the thymus were subjected to hematoxylin and eosin (H&E) staining for histological evaluation. The slides were visualized using an optical microscope (ZIESS, Oberkochen, Germany). Morphometric analysis was performed to objectively measure alterations in the cortical and medullary sizes of the thymus. The ratio of cortex to medulla was calculated using the ImageJ image processing and analysis software (Bethesda, MD).

### Immunohistochemical (IHC) Analysis

Tissue sections of the thymus were dewaxed, rehydrated, and then antigen-retrieved in Trilogy (Cell Marque, AR) at 121 °C for 15 min. The sections were separately incubated with ice methanol containing 3% H_2_O_2_ and blocked in PBS with 2.5% goat serum to reduce the endogenous peroxidase activity and nonspecific reactions. The slides were incubated with antimouse IL-7 antibody (ThermoFisher, MA; OriGene, MD) at 4 °C overnight and then treated with ImmPRESS HRP Goat Anti-Rabbit Polymer (Vector Laboratories, Burlingame, CA) for 30 min. For visualization, slides were incubated with the horseradish peroxidase (HRP) substrate 3,3′diaminobenzidine for 3–7 min followed by hematoxylin counterstaining for 1 min in the dark. The dark-brown positive signals were counted manually. All photos were captured using an optical microscope (ZIESS, Oberkochen, Germany).

### Preparation of Thymus Protein Extracts and Western Blotting

Following the manufacturer’s instructions, the thymus was harvested from each experimental group and homogenized in a mammalian cell lysis buffer supplemented with protease inhibitors (GoldBio, MO). Total protein was extracted and quantified using the bicinchoninic acid (BCA) protein assay (ThermoFisher, MA). Equal amounts of protein (40 μg) were loaded on 10–12% SDS-PAGE gels (polyacrylamide gel electrophoresis) and subsequently transferred to a poly(vinylidene fluoride) (PVDF) membrane using the protocol of the Trans-Blot Turbo Transfer System (Bio-Rad, München, Germany) with Trans-Blot Turbo RTA Transfer Packs. After being blocked with EveryBlot Blocking Buffer at room temperature, the PVDF membranes were incubated overnight at 4 °C with primary antibodies against IL-7 (ThermoFisher), IL-7R (Origene), SCF (ThermoFisher), c-KIT, FOXO3A, GABPA (Genetex, Hsinchu, Taiwan), LYL1 (ThermoFisher), or β-actin (Genetex). After washing with TBST buffer, the membranes were incubated for 1 h at room temperature with HRP-conjugated goat antirabbit secondary antibodies (Bio-Rad, CA). Following three washes with TBST, the protein bands were visualized using an enhanced chemiluminescence (ECL) detection system (Bio-Rad) on a Bio-Rad ChemiDoc XRS+ System. Densitometric analysis of the bands was performed using Bio-Rad Image Lab software with β-actin serving as loading control.

### Statistical Analysis

Statistical analyses were conducted using GraphPad Prism version 9 software (GraphPad Software, Inc., La Jolla, CA). Data were expressed as the mean ± standard error mean (SEM) and were determined for each treatment group in individual experiments. To assess the impacts of FPN compared to the VH group, statistical analyses were performed using one-way analysis of variance (ANOVA) followed by Dunnett’s two-tailed *t* test. A *p*-value <0.05 was considered statistically significant. All analyses were carried out in a blinded fashion.

## Results

### FPN Affected Body Weight, Thymus Index, and Population of Thymocytes *In Vivo*

After being administered corn oil (VH) or three different dosages of FPN (1, 5, 10 mg/kg) for a total of seven doses, mice showed no clinical signs or mortality. However, the group that received 10 mg/kg of FPN exhibited a remarkable weight reduction, and all FPN-treated groups displayed a significant decrease in the thymus index compared to that of the VH group. During FPN administration, the population of CD4^+^, CD8^+^, and CD4^+^/CD8^+^ double-negative (DN) thymocytes significantly increased, while the proportion of CD4^+^/CD8^+^ double-positive (DP) cells decreased. Notably, the T-cell receptor (TCR) subunit percentages remained unaltered (Table 2). To avoid potential misinterpretations caused by evaluating FPN toxicity based on percentage changes alone, we also calculated absolute thymocyte counts and the number of different subsets of thymocytes. Our analysis revealed that in the 10 mg/kg FPN group, the absolute number of CD4^+^/CD8^+^ DP thymocytes was significantly reduced, aligning with the slight decrease in the absolute number of CD4^+^ and CD8^+^ single-positive (SP) thymocytes. There was a slight increase in the number of CD4^–^/CD8^–^ DN thymocytes. Interestingly, although the proportion of TCR α/β^+^ and TCR γ/δ^+^ cells remained unchanged, the absolute cell numbers of TCR α/β^+^ and TCR γ/δ^+^ cells were decreased in FPN-treated groups.

**Table 2:** Effects of FPN on Body Weight, Thymus Index, and Cellularity of Thymocytes^a^,^b^,^c^,^d^

			fipronil (mg/kg)		
	NA	VH	1	5	10
body weight					
day 1	17.5 ± 0.72	17.19 ± 0.54	17.52 ± 0.6	17.58 ± 0.54	17.01 ± 0.47
day 10	21.07 ± 0.64	20.24 ± 0.46	20.69 ± 0.44	20.56 ± 0.35	18.77 ± 0.25*
thymus index					
index^d^	3.82 ± 0.16	3.68 ± 0.13	3.26 ± 0.14*	3.06 ± 0.1*	2.81 ± 0.2*
total number (×10^8^)^d^	1.392 ± 0.58	1.464 ± 0.74	1.217 ± 0.78	1.155 ± 0.64*	0.941 ± 0.6*
thymus cellularity (%)					
CD4^+^	13.54 ± 0.32	12.61 ± 0.48	15.02 ± 0.17*	16.01 ± 0.24*	15.58 ± 0.31*
CD8^+^	5.80 ± 0.35	6.06 ± 0.47	7.58 ± 0.11*	7.25 ± 0.20*	6.79 ± 0.28
CD4^+^/CD8^+^	76.61 ± 0.71	78.24 ± 0.93	73.84 ± 0.76*	70.98 ± 1.04*	71.18 ± 1.21*
CD4^–^/CD8^–^	3.70 ± 0.19	3.53 ± 0.15	3.73 ± 0.12	4.74 ± 0.28*	5.45 ± 0.32*
TCR α/β^+^	34.58 ± 6.6	35 ± 7.08	34.85 ± 6.52	33.01 ± 7.26	34 ± 6.46
TCR γ/δ^+^	0.59 ± 0.23	0.39 ± 0.08	0.3 ± 0.05	0.33 ± 0.07	0.4 ± 0.09
number of different subsets of thymocytes (×10^6^)^d^					
CD4^+^	17.94 ± 1.02	17.29 ± 1.34	18.28 ± 1.18	19.32 ± 1.22	15.85 ± 1.15
CD8^+^	7.685 ± 0.43	8.309 ± 0.64	9.228 ± 0.59	8.749 ± 0.55	6.908 ± 0.5
CD4^+^/CD8^+^	101.5 ± 5.79	107.2 ± 8.32	89.9 ± 5.82	85.66 ± 5.44	72.42 ± 5.27*
CD4^–^/CD8^–^	4.902 ± 0.27	4.84 ± 0.37	4.541 ± 0.29	5.72 ± 0.36	5.545 ± 0.4
TCR α/β^+^	45.81 ± 2.61	47.99 ± 3.72	42.42 ± 2.75	39.83 ± 2.53	34.59 ± 2.52*
TCR γ/δ^+^	0.781 ± 0.044	0.5347 ± 0.041	0.365 ± 0.023*	0.398 ± 0.025*	0.407 ± 0.029*

a Data were expressed as mean ± SEM of triplicate samples pooled from four independent experiments (*N* = 20/group). *p < 0.05 as compared with the VH group.

b Thymus index was calculated as the thymus weight (mg) per body weight (g). Data are expressed as mean ± SEM of 20 samples pooled from 4 independent experiments.

c Thymocytes were prepared as described in the Materials and Methods section. The percentage of CD4^+^/CD8^+^, TCR α/β^+^, and TCR γ/δ^+^ cells was determined by flow cytometry.

d Percent values and total number of thymus were used to calculate the total number of each cell population in the thymus.

### FPN Leads to a Dose-Dependent Reduction in Thymocyte Numbers in Mice

**Figure 2:**
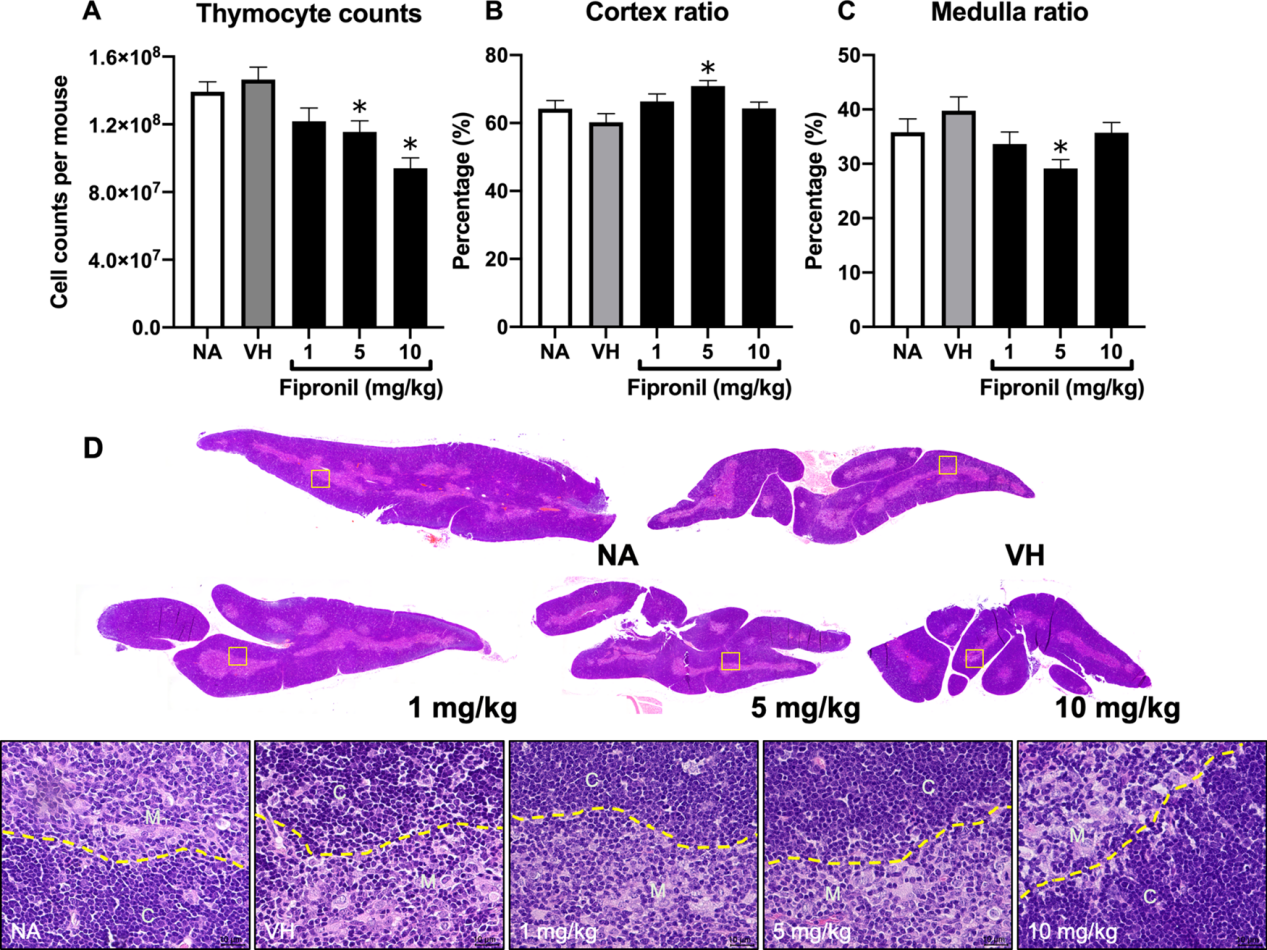
Reduction of thymocyte counts, thymus size, and medulla/cortex ratio by FPN. (A) Total thymocyte counts were isolated from each mouse and expressed as means ± SEM. (B, C) Ratio of cortex/medulla was quantified using ImageJ software as described in Materials and Methods. The cortex/medulla ratio was expressed as the mean ± SEM of thymus sections pooled from four independent experiments. *p < 0.05 as compared with the VH group. (D) Representative H&E-stained histological images of thymus sections in each treatment group were shown (spliced from original magnification 100×). The lower panels are enlarged images of the area of yellow boxes (original magnification, 400×). Areas marked by the dashed line distinguish the cortex or medulla regions.

We subsequently investigated the impact of FPN on the total number of thymocytes. In the FPN treatment groups, we observed a dose-dependent reduction in the total number of thymocytes, with a significant decrease noted in the 5 and 10 mg/kg groups. In the highest dose group (10 mg/kg), the total number of thymocytes reduced to only 64.3% (about 9.41 × 10^7^ cells/thymus) compared with the VH control group (about 1.46 × 10^8^ cells/thymus), representing a 35.7% decrease (Figure 2A).

### Effects of the Cortex/Medulla Ratio and Thymus Atrophy

Histological analysis of thymic H&E sections included an assessment of the cortex/medulla ratio and overall thymic area ratio. The results revealed a significant decrease in the medullary proportion and a corresponding increase in the cortical proportion in the 5 mg/kg group (Figure 2B,C). Mice treated with 10 mg/kg FPN exhibited a marked decrease in thymocyte numbers and the overall thymic area proportions (the overall area reduced to approximately 80.06% compared to the VH group) without alteration of the cortex/medulla ratio.

### FPN Significantly Attenuated mRNA Expression of IL-7 in the Thymus

**Figure 3:**
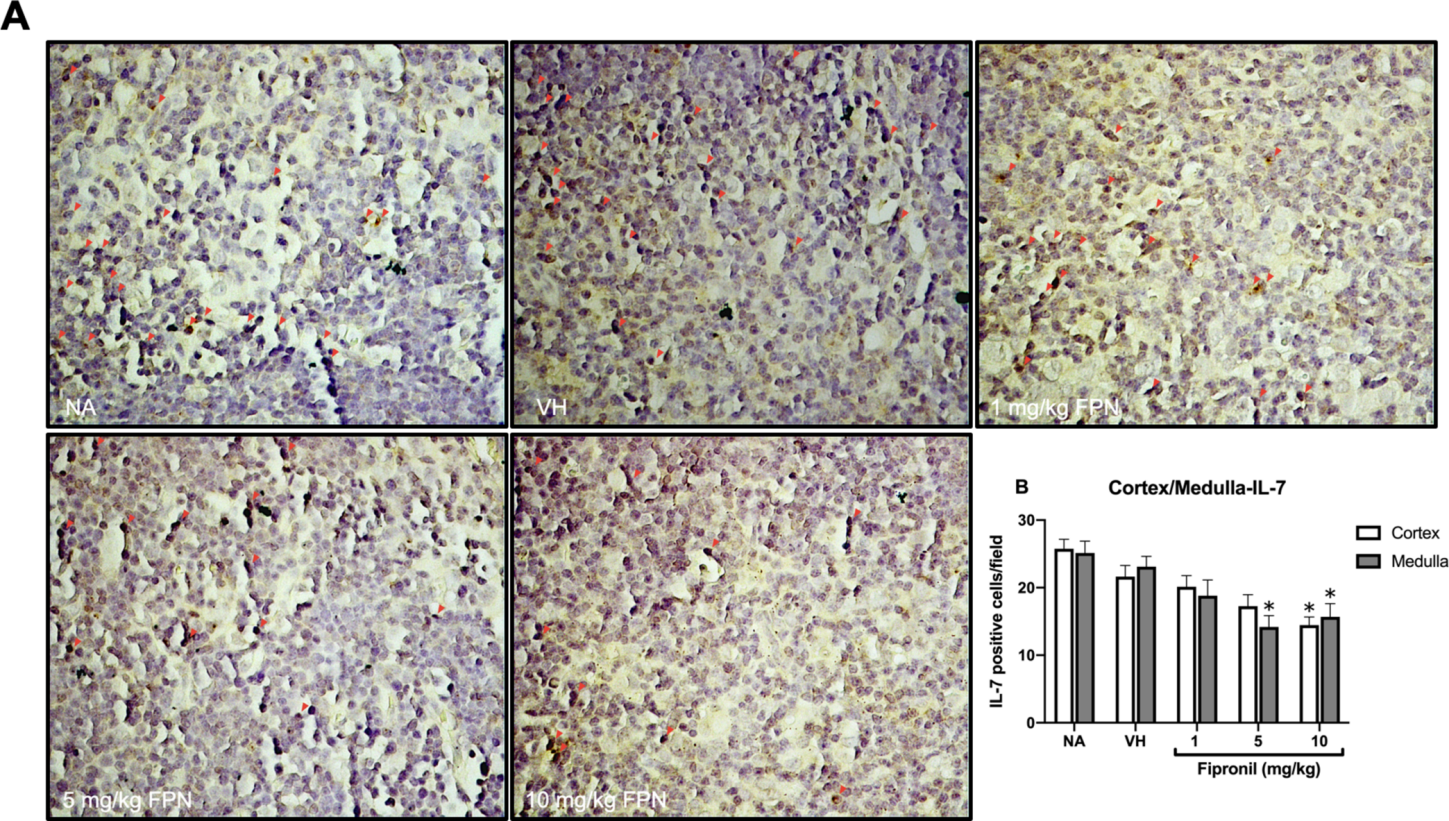
Fipronil significantly decreased IL-7-positive cells in the thymus. (A) Representative immunohistological images of thymus sections in each treatment group were shown (original magnification, 400×). Arrows indicate IL-7^+^ cells with red signals. (B) Quantified data for the number of IL-7 positive cells from the cortex or medulla area were expressed as the mean ± SEM of 20 samples per group (*N* = 20/group). *p < 0.05 was significant compared to the VH group.

The level of IL-7 mRNA was significantly decreased in the FPN treatment groups. Therefore, the IL-7 protein levels were further confirmed by immunohistochemistry. The analysis reveals that the number of IL-7 positive cells was significantly attenuated in both the cortex and medulla area at high-dose FPN treatment groups (Figure 3).

### FPN Significantly Decreased mRNA Expression of Transcription Factors of T-Cell Lineage and IL-7 Signaling in the Thymus

**Figure 4:**
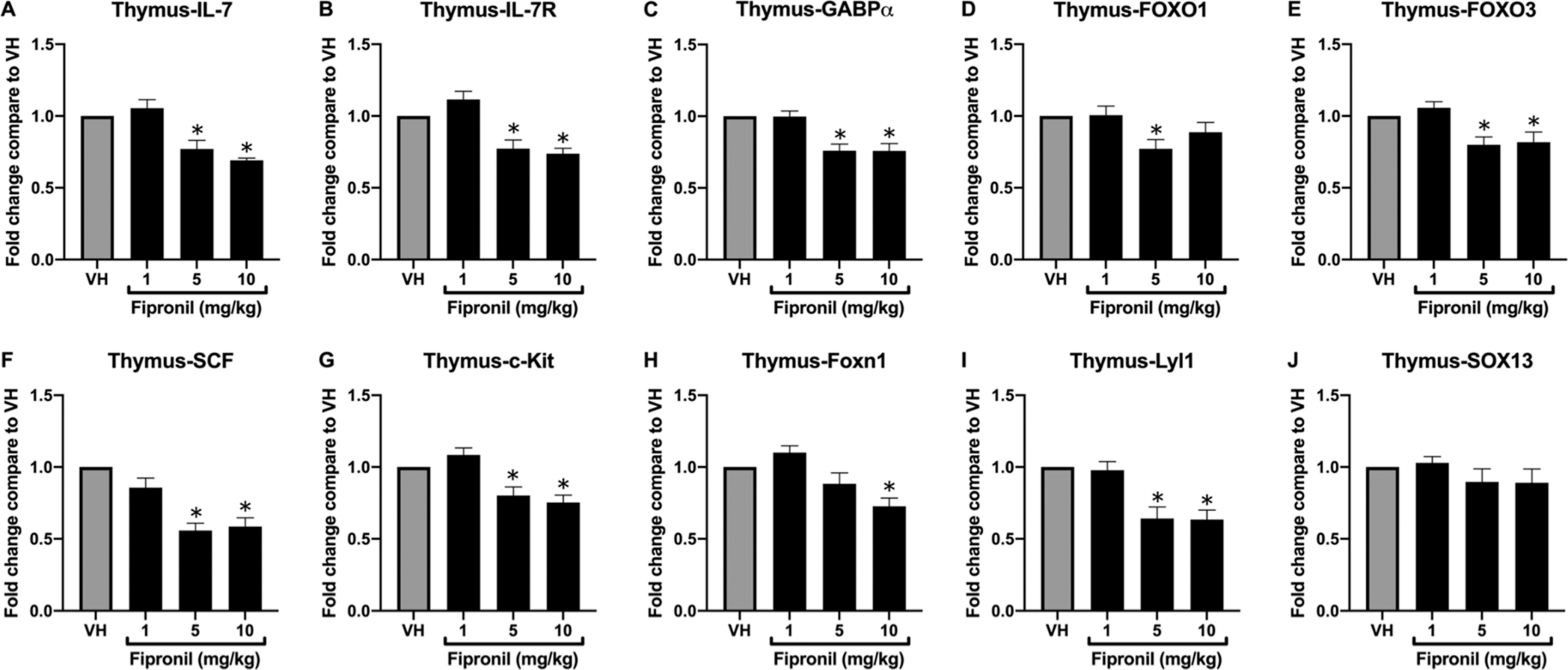
Fipronil significantly decreased the mRNA expression of transcription factors of T-cell lineage and IL-7 signaling in the thymus. The total RNA of the thymus harvested from different treatment groups was extracted to detect the mRNA expression of transcription factors of T-cell lineage and IL-7 signaling by qPCR. The expression level of *HPRT* was used as the control for semiquantification. Results were expressed as the mean ± SEM pooled from four independent experiments with technological duplication in each group (*N* = 20/group). *p < 0.05 was significant compared to the VH group.

The total mRNA of the thymus tissues was directly extracted for qPCR detection. The analysis reveals a significant decrease in the mRNA expression levels related to the IL-7 signaling pathway, including IL-7, IL-7R, GABPα, FOXO1, and FOXO3. Furthermore, in the key transcription factors essential for T-cell maturation and development, a downward trend in the expression levels of Foxn1, Lyl1, SCF, and c-Kit was observed (Figure 4).

### Reduction of T-Cell Lineage Transcription Factors and IL-7 Signaling-Associated Proteins in the Thymus by FPN

**Figure 5:**
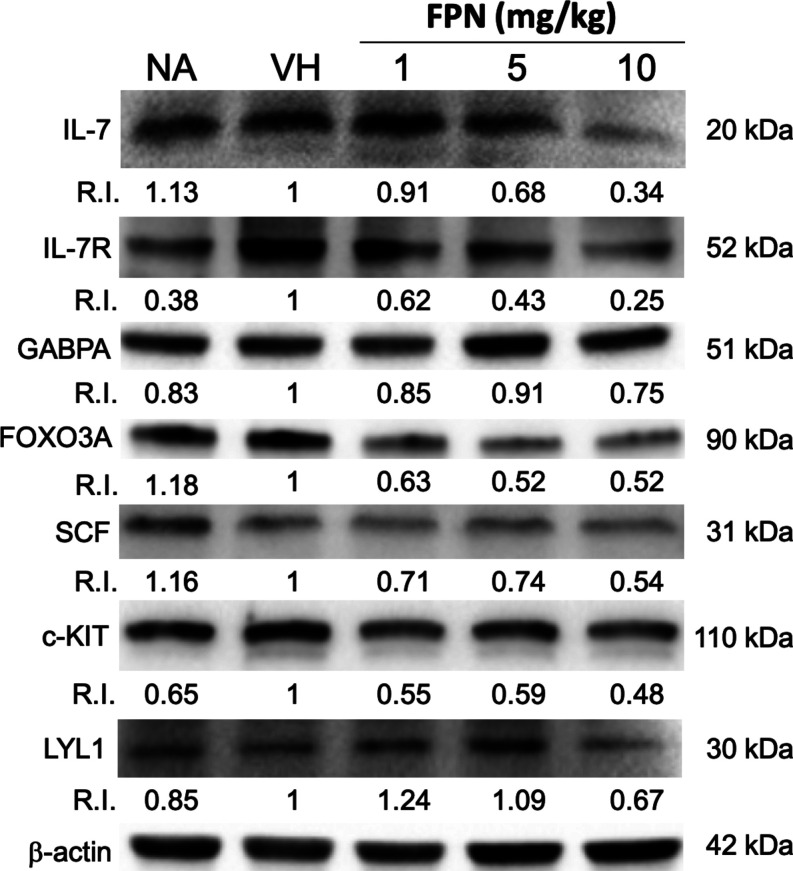
Fipronil markedly decreased T-cell lineage transcription factors and IL-7 signaling-associated proteins in the thymus. The total protein of the thymus harvested from different treatment groups was extracted to detect the protein expression of transcription factors of T-cell lineage and IL-7 signaling by Western blotting. The expression level of β-actin was used as the loading control for semiquantification. The different protein/β-actin ratio in the treatment group was divided by the protein/β-actin ratio in VH as the relative intensity (RI). The result was representative of three independent experiments (*N* = 9/group).

Given the significant reduction in the expression of genes related to IL-7 signaling, we further investigated whether protein levels in the thymus were similarly affected by FPN. Consistent with the mRNA results, the protein levels of IL-7, IL-7R, GABPA, FOXO3A, SCF, c-KIT, and LYL1 were reduced to varying degrees when normalized to the intensity of the VH group (Figure 5).

### FPN Significantly Decreased mRNA Expression of IL-7R, *SCF, GABP*α, *Lyl*1, and *SOX*13 in ConA-Stimulated Thymocytes

**Figure 6:**
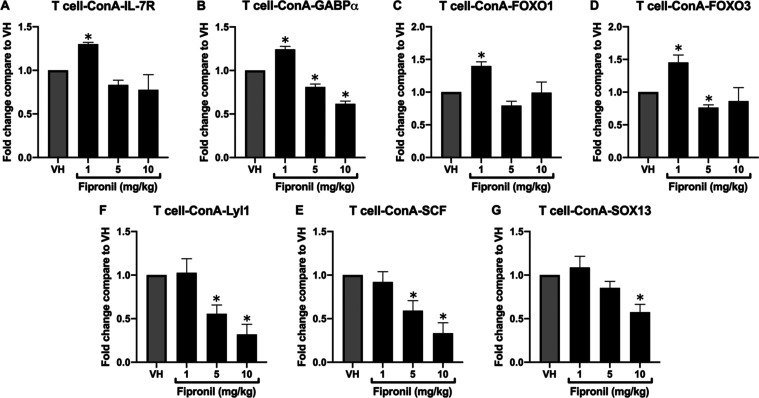
Fipronil significantly decreased mRNA expression of *Lyl*1, SOX13, SCF, IL-7*R*, and *GABP*α in ConA-stimulated thymocytes. The total RNA of thymocytes (6 × 10^6^ cells/mL) harvested from different treatment groups stimulated by ConA was extracted to detect the mRNA expression of *Lyl*1, *SOX*13, *SCF*, and *IL-*7 receptors by qPCR. The expression level of *HPRT* was used as the control for semiquantification. The expression level of *HPRT* was used as the control for semiquantification. Results were expressed as the mean ± SEM pooled from four independent experiments with technological duplication in each group (*N* = 20/group). *p < 0.05 was significant compared to the VH group.

The primary thymocytes were isolated and stimulated by ConA for 24 h to further assess mRNA expression of IL-7 signaling genes and transcription factors of the T-cell lineage, which are essential for the development and differentiation of T cells into mature T cells with specific functions in the immune system. The results showed that the mRNA expression of, *IL-*7 receptor, *SCF*, *GABP*α, Lyl1, and SOX13 was notably decreased in the high dose of FPN compared to the VH control (Figure 6).

### Differential Effects of FPN on the Production of IL-2, IL-4, and IFN-γ *Ex Vivo*

**Figure 7:**
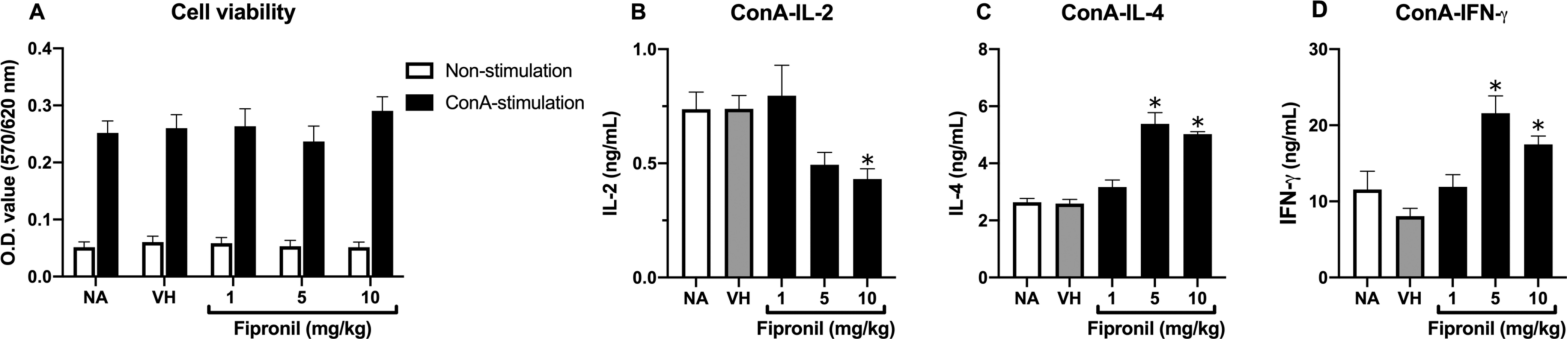
Fipronil exhibited an increase in IL-4 and IFN-γ and a decrease in IL-2 production stimulated by ConA. Thymocytes (6 × 10^6^ cells/mL) were prepared from each group of mice and cultured in the absence or presence of concanavalin A (ConA; 5 μg/mL) for 48 h. The metabolic activity of cells was determined by an MTT assay, and the level of IL-2, IL-4, and IFN-γ in the supernatants was measured by ELISA. Data was expressed as the mean ± SEM of quadruplicate cultures and representative of four independent experiments (*N* = 20/group). *p < 0.05 was significant compared to the VH group.

The cytotoxic effect of FPN on thymocytes was assessed by using the MTT assay. We assessed the cytokine levels under ConA stimulation. ConA induces T-cell mitosis by binding to mannose and glucose residues on glycoproteins. These bindings lead to the cross-linking of cell surface receptors, activation of signaling pathways, calcium influx, and subsequent activation of transcription factors. These factors drive cell cycle progression, ultimately resulting in mitosis. There was a significant decrease in the production of IL-2, accompanied by an increase in both IL-4 and IFN-γ by FPN (Figure 7).

## Discussion

Extensive research has delved into the neurotoxic, reproductive, and cytotoxic effects induced by fipronil,^10,42−44^ but studies of immunotoxicity specifically related to lymphocyte function are lacking. Administration of fipronil induces inflammatory responses, demonstrating immunotoxic effects in Wistar rats. After exposure to FPN for 30 days, the general architecture of the thymus was changed with the pale medulla and thick cortex, indicating the developing T lymphocytes were trapped in the outer cortex. Additionally, the cortico-medullary junction appeared indistinct, with obvious aggregation of proteinaceous eosinophilic cells and numerous phagocytosed apoptotic bodies present in the medulla.^38^ However, none of these studies have comprehensively investigated the effects of FPN on the T-cell functionality and its underlying mechanisms. In our previous study, we determined the immunotoxic effects of FPN on T-cell-mediated immune responses and demonstrated the interference of FPN with mature T-cell function.^40^ In this study, we aim to study the deleterious impacts of FPN on thymic development and T-cell lineage commitment, thereby the young age BALB/c mice (4-week-old) were applied in this study.^45^

The thymus is one of the primary lymphoid organs that generates self-tolerant and immunocompetent T lymphocytes. Thymopoiesis is an intricate process that involves the maturation and differentiation of thymocytes (immature T cells) into functional and diverse T-cell subsets, which are tightly regulated by various signaling pathways, transcription factors, and interactions with stromal thymic stromal cells. The functionality of the thymus will remain developed for the whole lifetime, especially following hematopoietic cell stress, despite that thymus size will decline with age, named thymic involution (age-related atrophy).^28,46^ Human thymic involution is thought to begin at 1 year of age, whereas murine thymic involution peaks at 4 weeks of age and gradually decreases thereafter.^47^ The impacts of thymic atrophy are most profound in clinical conditions that result in severe loss of peripheral T cells that could contribute to a reduction of pathogen defense, a high incidence of autoimmune responses, and the attenuation of immunosurveillance.^48,49^ The current data demonstrated that FPN induced thymus atrophy and downregulated important signaling pathways crucial for thymocyte selection and differentiation. To the best of our knowledge, this study represents the first investigation of the immunotoxic effects of FPN on thymic development through the dysregulation of essential development transcription factors and the IL-7-associated genes following short-term oral exposure.

Our results demonstrated that despite exposure to FPN (1–10 mg/kg), seven doses did not elicit severe clinical symptoms and there were significant changes in both thymic index and thymocyte cellularity. Intriguingly, the thymus index was notably decreased in each FPN treatment group, while the body weight gain was significantly decreased only in the 10 mg/kg FPN groups on day 10. These results suggest that the dosage of 10 mg/kg of FPN may have more pronounced toxicity in mice. The slight decrease in body weight could be linked to oxidative stress provoked by FPN (the administered dosage was ≤9.7 mg/kg (1/10 LD_50_)).^12^ In parallel with the decline of the thymus index, the total number of thymocytes isolated from each mouse of FPN-treated groups significantly decreased in a dose-dependent manner. Histological analysis showed a reduction of the medulla–cortex ratio, suggesting atrophy in the medulla area of the thymus. In the 10 mg/kg FPN group, although the cortex/medulla ratio did not exhibit the most substantial difference, the total size of the thymus retained only about 80% consistent with a significant reduction of 35% of the total number of thymocytes, indicating an overall atrophy of the thymus.

Furthermore, based on the alteration of thymus cellularity in FPN-treated mice, there is a clear imbalance in the ratio of CD4/CD8 thymocyte population. Distribution confusion of the CD4/CD8 subsets was similarly verified in the absolute number from each cell population. The immature thymocytes were blocked in the CD4^–^/CD8^–^ double-negative (DN) stage, leading to the deficiency of the CD4^+^/CD8^+^ double-positive (DP) T-cell population. This data suggested that FPN-induced cell arrest in the DN stage without successfully transitioning to the DP stage may be due to the toxic effect of FPN on the early stage of thymocyte development. Interestingly, the proportion of CD4 and CD8 SP thymocytes remained unchanged or slightly increased during the FPN exposure. We speculate that thymocyte development led to an increase in the number of mature CD4 and CD8 SP thymocytes, which may be a compensatory response to early developmental disruptions. Collectively, these findings suggest the potential immune toxic effects of FPN on the thymus, accompanied by induction of thymus atrophy and disruption of DP thymocyte expansion.^50^ The pre-TCR is expressed during the DN3 stage following TCRβ rearrangement, and subsequent TCRα rearrangement leads to the development of DP T cells. After the positive and negative selection processes, these cells are converted into CD4 or CD8 SP thymocytes. TCR integrity and signaling are crucial for thymocyte proliferation, development, and maturation.^51,52^ Our results showed that the number of TCR α/β^+^ and TCR γ/δ^+^ cells was decreased in FPN-treated groups, indicating that FPN may downregulate the expansion of TCR α/β^+^ and TCR γ/δ^+^ cells during development.

The dynamic regulation of IL-7 signaling profoundly influences thymus and T-cell development. IL-7 is a fundamental requirement for each lymphocyte during its initial developmental stage.^53−56^ During the DN stage, immature thymocytes experience their initial interaction with IL-7, which is essential for the survival of DN thymocytes to progress into the subsequent developmental stage.^57^ IL-7Rα expression commences upon entry into the DN2 stage.^58^ Experimental depletion of IL-7 through injections of IL-7 or IL-7R antibodies *in vivo* resulted in a profound reduction in overall thymocyte numbers (>99%).^59−61^ Additionally, genetic deletion of IL-7, IL-7R, or proximal signaling molecules of IL-7R led to a severe defect in thymopoiesis and a developmental block at the DN3 stage. These data strongly support the indispensability of IL-7 for the survival of post-β-selection DN thymocytes.^59,61−63^ Furthermore, initiating a STAT5-dependent opening of the TCR γ-chain locus for TCR rearrangement in γδ T-cell development also necessitates IL-7 signaling.^54,55^ This process has also been proposed for the TCR β-chain locus during αβ T-cell development.^56^ In our results, a series of genes and proteins related to IL-7 are significantly decreased by FPN exposure, suggesting that FPN-mediated immunodeficiency effects may be closely associated with the dysregulation of the IL-7 signaling pathway. Oral administration of tributyltin acetate resulted in a decrease in CD4 and CD8 SP T-cell populations and blocked the thymocyte differentiation at the DP and DN stages by down-regulating IL-7 mRNA in thymic epithelial cells.^64,65^ During severe thymic atrophy induced by dexamethasone or irradiation, the regeneration of the thymus occurred through upregulation of IL-7 expression.^66^ Collectively, these data suggest that the regulation of IL-7 plays a crucial role in compensating for different chemical stimuli. Disruption of crosstalk between thymic epithelial cells and thymocytes may lead to a reduction in the level of mature T-cell development.

Several transcription factors have already been recognized, whose interaction and cross-regulatory network might be associated with the IL-7Rα expression. Such GA-binding protein (GABP), the Ets family transcription factor, has been identified as essential for the upregulation of IL-7Rα in immature DN thymocytes.^67^ Forkhead box O (*FOXO*) 1 transcription factor deficiency resulted in a severe defect in IL-7Rα expression.^68^*FOXO*1-deficient mice can develop a lethal inflammatory disorder and lead to an elevation in CD4 and CD8 single-positive (SP) thymocyte populations.^69,70^*FOXO*3*a*-deficient mice lead to mild lymphoproliferative syndrome and the formation of inflammatory lesions. Furthermore, *FOXO*3*a* deficiency will also develop a systemic and spontaneous autoimmune syndrome attributed to hyperactive NF-κB signaling in T cells.^71^ These pieces of evidence suggest that the well-regulation of the FOXO family will direct the normal development of thymocytes in different stages associated with *IL-*7*R* expression. In the present study, the expression of *IL-*7, IL-7R, *GABPα*, *FOXO*1, and *FOXO*3 was decreased in thymus tissues of 5 and 10 mg/kg FPN-treated groups. Interestingly, only *GABP*α was dose-dependently decreased in ConA-stimulated thymocytes isolated from FPN-treated mice. After ConA stimulation, the expression of *IL-*7*R*, *FOXO*1, and *FOXO*3 by thymocytes was not altered in high-dose FPN groups, while it exhibited a slight or even significant increase in the 1 mg/kg FPN group. We hypothesize that when mice are exposed to a lower dosage of FPN, a defense mechanism may be activated to counteract the potential immunotoxicity induced by FPN. However, the defense mechanism may become ineffective at higher dosages. Another speculation may be that these genes expressed by other parenchymal cells, such as thymic epithelial cells (TECs), in the thymus are more sensitive to FPN treatment than thymocytes.

A decrease in the IL-7 positive signals has been observed in high-dose groups (5 and/or 10 mg/kg of FPN) by IHC staining. Meanwhile, the Western blot results also showed a downward trend in the levels of key functional proteins, including IL-7, IL-7R, SCF, c-KIT, GABPA, FOXO3A, and LYL1, which is consistent with the observed mRNA trends. Given the reduction in IL-7 positive cells and the significant downregulation of IL-7 signaling-related genes and proteins, the proliferation, development, and T-cell lineage commitment are markedly impaired, ultimately resulting in immunodeficiency. Accordingly, we hypothesized that FPN progressively reduced expression levels of genes and proteins associated with IL-7 signaling, which was coregulated by downstream transcription factors. Therefore, the decline in these genes and proteins mainly results in a reduction in thymocyte numbers and a disruption of the thymic microenvironment. In our results, the decreased levels of *IL-*7 and *FOXO*1 might also contribute to the slight activation of CD4 transcription, promoting an increased population of CD4^+^ T cells.^69,72^ Dysregulation of the CD4/CD8 ratio can lead to various immune dysregulations, impacting adaptive immune responses and potentially leading to immunodeficiency or autoimmune disorders.^48,49^ Additionally, we propose that the immunotoxic effects of FPN may involve the regulation of apoptotic pathways by FOXO, such as the upregulation of Fas ligand (FasL) or tumor necrosis factor-related apoptosis-inducing ligand (TRAIL), as well as pro-apoptotic/preapoptotic adjustment by Bcl-2 family member (Fu and Tindall, 2008). This speculation requires further research.

Besides IL-7 signaling genes, several transcription factors are essential to thymic development. The full growth and differentiation of TECs have relied on *Foxn*1 activation. *Foxn*1 also promotes the downstream transcription of genes implicated in thymus organogenesis.^73^ In the absence of *Foxn*1 expression, the intrathymic lymphopoiesis of affected patients is completely blocked,^74,75^ leading to severe primary T-cell immunodeficiency,^76−78^ which is also observed on the *Foxn*1^–/–^ mice model.^79^*Lyl*1 has been recognized as a critical component responsible for orchestrating lymphoid specification in multipotent bone marrow progenitors. Additionally, *Lyl*1 plays a vital role in sustaining the survival and expansion of thymic cell progenitors, particularly during the crucial stages of pro-T-cell expansion. Lack of *Lyl*1 in early T lineage progenitors and DN thymocyte progenitors exhibits in increased apoptosis, blocked differentiation, and impaired population expansion.^37,80^ Stem cell factor (*SCF*) is produced by stromal cells and interacts with its ligand *c-Kit* expressed by DN thymocytes. Proper coordination between *SCF*, *c-Kit*, and IL-7 signaling pathways is essential for the progression of thymopoiesis and the production of functional T cells in the thymus.^34,35^ In the present results, the mRNA expression of these transcription factors associated with T-cell progenitors’ differentiation, survival, and expansion were significantly reduced by FPN. These findings highlight the immunotoxic effects of FPN, presumably resulting in the disorder of thymopoiesis. As FPN exhibits persistent bioaccumulation of a prolonged presence of FPN and its metabolites in the body,^38,81−83^ exposure to FPN may contribute to a disturbance in thymic functionality and homeostasis.

Under the ConA stimulation *ex vivo*, the secretions of IL-2 were decreased, and the IL-4 and IFN-γ were increased, suggesting the disturbance of thymocyte function by FPN. The dysregulation of cytokine production can play a pivotal role in the development of immunodeficiency syndromes and various T-cell lymphoproliferative disorders. IL-2 regulates T-cell growth, proliferation, differentiation, and the maturation of different subsets of T cells in the thymus.^84^ The absence of IL-2 can lead to conditions such as anemia, abnormal lymphoproliferation, and an inflammatory bowel disease akin to ulcerative colitis.^85^ IL-4 may also influence T-cell maturation in the thymus. *In vivo*, overexpression of IL-4 has been associated with a reduction in the total number of immature thymocytes, accompanied by an increase in the number of mature CD8^+^ thymocytes, mirroring the observed trends in the cellularity of CD8^+^.^86,87^^86,87^ Similar outcomes have been reported in IFN-γ transgenic mice, where CD4 or CD8 single-positive T-cell populations were elevated.^88^ Additionally, the increased secretion of IFN-γ might be mediated by the decreased levels of the *FOXO*3*a* gene.^89^ Our data showed a diminishment of IL-2 production with a disturbance of Th1 or Th2 cytokine production. We speculate that the thymus might be compensating for FPN-induced toxicity by accelerating the maturation process to maintain the SP thymocyte population. However, this accelerated maturation might lead to functional dysregulation in these rapidly matured thymocytes.

Survival, maturation, and trafficking of T cells in the thymus are regulated by the thymic hypersensitivity to glucocorticoids (GC).^90^ High GC levels can induce T-cell apoptosis and have an immunosuppressive effect on T cells, potentially affecting T-cell selection and causing thymus atrophy.^27,91−93^ Treatment of Wistar rats with 1/20 LD_50_ of FPN for 6 weeks significantly induced higher serum corticosterone levels (approximately 141.31 pg/mL),^94^ which is the major stress hormone controlled by corticotropin-releasing hormone and adrenocorticotropic hormone in the Hypothalamus–Pituitary–Adrenal (HPA) axis. This potential immunotoxic mechanism of GC is related to oxidative damage.^94−96^ GC acts by binding to the glucocorticoid receptor (GR), which is expressed by all thymocytes during their development, albeit at different levels in each CD4/CD8 subset 94,95. Despite CD4^+^/CD8+ with the lowest GR level, they exhibit the highest sensitivity to GC-induced apoptosis 88,89. As FPN may induce corticosterone in serum, the elevated cortisol effects on thymus atrophy may be one of the potential mechanisms involved in FPN-induced thymus atrophy. Further studies are needed to evaluate how FPN regulates corticosterone levels within the thymus and to elucidate their roles in thymocyte development.

Although the acceptable daily intake (ADI) of FPN is 0.0002 mg/kg, a very conservative safety threshold for risk management of chronic exposure, in the real world, accidental or occupational exposure may occur at high doses. Cam et al. summarized different FPN exposure conditions and the serum level of the major FPN metabolite, fipronil sulfone, in human cases. In a self-poisoning case, the maximum fipronil and fipronil sulfone levels could reach 3.74 μg/mL.^25^ In comparison to a pharmacokinetics study, the plasma levels of FPN or fipronil sulfone concentration reached around 0.6 and 1.2 μg/mL, respectively, after a single oral dose of FPN (10 mg/kg).^83^ As previous toxicology studies applied similar or higher doses to elucidate the effects of FPN on different biological systems, the present study included 1/100 to 1/10 of the oral LD_50_ (1–10 mg/kg) to minimize the risk of acute toxicity and mortality while still inducing subchronic toxic effects to demonstrate dose-dependent effects of FPN on thymopoiesis.^42,97^ Collectively, our study may still be valid to provide scientific evidence for further evaluation of the immunotoxicity of FPN due to intentional or unintentional exposure.

## Conclusions

This study demonstrated that oral exposure to FPN for seven doses induced thymic atrophy and altered both the thymic cellularity and absolute thymocyte numbers across different subpopulations. These immunotoxic effects are attributed to the dysregulation of genes and proteins involved in IL-7 signaling transduction as well as the impaired functionality of crucial transcription factors essential for thymocyte survival, thymic development, and T-cell lineage commitment. This study may open an avenue to investigate the immunotoxic effects of FPN on T-cell development. Combined with our previous research that FPN disturbed antigen-specific T-cell responses *in vivo*, the cumulative immunotoxicity of FPN needs to be given more attention.

## Data Availability

The original data employed or analyzed in this present study can be obtained from the corresponding author upon making a reasonable request.

## Abbreviations

***FPN***: fipronil
***ConA***: concanavalin A
***TEC***: thymic epithelial cells
***ETP***: early T lineage progenitors
SCF: stem cell factors
c-Kit: tyrosine-protein kinase KIT
GABPα: GA-binding protein α
FOXO: forkhead box-O
*Foxn*1: forkhead box protein N1
*Lyl*1: lymphoblastomic leukemia 1
*SOX*13: SRY-box transcription factor 13
